# TEPP (*O*,*O*,*O*,*O*-tetraethyl­pyrophosphate): MAK Value Documentation, addendum – Translation of the German version from 2024

**DOI:** 10.34865/mb10749e11_2ad

**Published:** 2026-06-30

**Authors:** Andrea Hartwig, MAK Commission

**Affiliations:** 1 Institute of Applied Biosciences, Department of Food Chemistry and Toxicology, Karlsruhe Institute of Technology (KIT) Adenauerring 20a, Building 50.41 76131 Karlsruhe Germany; 2 Permanent Senate Commission for the Investigation of Health Hazards of Chemical Compounds in the Work Area, Deutsche Forschungsgemeinschaft, Kennedyallee 40, 53175 Bonn, Germany. Further information: Permanent Senate Commission for the Investigation of Health Hazards of Chemical Compounds in the Work Area | DFG

**Keywords:** TEPP (O,O,O,O-tetraethyl­pyrophosphate), insecticide, pesticide, toxicity, evaluation, acetylcholinesterase inhibitor

## Abstract

TEPP (*O*,*O*,*O*,*O*-tetraethylpyrophosphate) [107-49-3] is used as an insecticide and acaricide. In the European Union it has never been approved and it is not approved in Germany since 1967. The previous MAK value documentation and addendum do not reflect the current data situation of the substance. The MAK Commissiondecided that a new evaluation is not of high priority. The MAK value and the other classifications are therefore suspended and the substance is listed in the Section II c of the List of MAK and BAT Values for substances no longer evaluated.

**Table:** 

**MAK value**	**see Section II c of the List of MAK and BAT Values**
**Peak limitation**	**–**
	
**Absorption through the skin**	**–**
**Sensitization**	**–**
**Carcinogenicity**	**–**
**Prenatal toxicity**	**–**
**Germ cell mutagenicity**	**–**
	
**BLW (2023)**	**reduction of the acetylcholinesterase activity in erythrocytes to 70% of the reference value^a)^**
	
Synonyms	diphosphoric acid tetraethyl ester
Chemical name (IUPAC)	diethoxyphosphoryl diethyl phosphate
CAS number	107-49-3
Molar mass	290.19 g/mol (IFA 2023)
Melting point	0 °C (IFA 2023)
Boiling point	> 170 °C (decomposes) (IFA 2023)
Density at 20 °C	1.185 g/cm^3^ (IFA 2023)
Vapour pressure	< 0.001 hPa (IFA 2023)
log K_OW_	–
Solubility	miscible with water, decomposes (IFA 2023)
**1 ml/m^3^ (ppm) ≙ 12.04 mg/m^3^**	**1 mg/m^3^ ≙ 0.083 ml/m^3^ (ppm)**

^a)^ The BLW (biological guidance value) is derived as the ceiling value because of acute toxic effects.

Note: The substance can occur simultaneously as vapour and aerosol.

This addendum was prepared because the previous evaluations no longer reflect the data currently available for the MAK value and for the designations and classifications of the substance. TEPP (*O*,*O*,*O*,*O*-tetraethylpyrophosphate) is an insecticide and acaricide that inhibits the cholinesterase in the blood and tissues. The biological guidance value (BLW) for acetylcholinesterase inhibitors (reduction of the acetylcholinesterase activity in erythrocytes to 70% of the reference value; Lewalter 1995; Weistenhöfer et al. 2024) therefore applies to TEPP. The BLW is derived as the ceiling value because of acute toxic effects. However, it was not investigated whether this is the most sensitive end point.

In 1958, a MAK value of 0.005 ml/m^3^ (0.06 mg/m^3^) was set and the substance was designated with an “H” (for substances which can be absorbed through the skin in toxicologically relevant amounts). In the addendum from 2002, TEPP was assigned to Peak Limitation Category II with an excursion factor of 2 (Greim 2002, available in German only; Henschler 1973, available in German only).

TEPP was never approved as a pesticide in the European Union (European Commission 2022; European Parliament and European Council 2009). It was approved in the former German Democratic Republic until 1967, but its use was never permitted in the Federal Republic of Germany (BVL 2010).

The previous evaluations (MAK value documentation and addendum) do not reflect the currently available data. However, a re-evaluation of the substance is not a priority. Therefore, the MAK value, the peak limitation and the “H” designation have been withdrawn and TEPP has been allocated to Section II c of the List of MAK and BAT Values (DFG 2022). This section lists substances for which the previous MAK values, designations and classifications have been withdrawn and which are no longer being reviewed at present.

## References

[ref_CK2UPRZX] BVL (Bundesamt für Verbraucherschutz und Lebensmittelsicherheit), editor (2010) Berichte zu Pflanzenschutzmitteln 2009. Wirkstoffe in Pflanzenschutzmitteln – Zulassungshistorie und Regelungen der Pflanzenschutz-Anwendungsverordnung. Volume 5/1. Basel: Springer Basel AG. https://www.bvl.bund.de/SharedDocs/Downloads/04_Pflanzenschutzmittel/bericht_WirkstoffeInPSM_2009.pdf?__blob=publicationFile&v=3, accessed 18 May 2022

[ref_I9LHTAFZ] DFG (Deutsche Forschungsgemeinschaft), editor (2022) List of MAK and BAT Values 2022. Maximum Concentrations and Biological Tolerance Values at the Workplace. Permanent Senate Commission for the Investigation of Health Hazards of Chemical Compounds in the Work Area, report 58. Düsseldorf: German Medical Science. https://doi.org/10.34865/mbwl_2022_eng

[ref_35SW4VLP] European Commission (2022) Tetraethyl pyrophosphate (TEPP). EU Pesticides Database (v.2.2). Active substances. https://ec.europa.eu/food/plant/pesticides/eu-pesticides-database/start/screen/active-substances/details/792, accessed 11 May 2022

[ref_PYFKDR5Q] European Parliament, European Council (2009) Regulation (EC) No 1107/2009 of the European Parliament and of the Council of 21 October 2009 concerning the placing of plant protection products on the market and repealing Council Directives 79/117/EEC and 91/414/EEC. OJ L. 309: 1-50

[ref_5NFDWGTQ] Greim H, editor (2002) TEPP (O,O,O,O-Tetraethylpyrophosphat). In: Greim H, Gesundheitsschädliche Arbeitsstoffe, Toxikologisch-arbeitsmedizinische Begründung von MAK-Werten. 34th issue. Weinheim: Wiley-VCH. Also available from https://doi.org/10.1002/3527600418.mb10749d0034

[ref_DSE2FK72] Henschler D, editor (1973) TEPP. In: Gesundheitsschädliche Arbeitsstoffe, Toxikologisch-arbeitsmedizinische Begründung von MAK-Werten. 2nd issue. Weinheim: VCH. Also available from https://doi.org/10.1002/3527600418.mb10749d0002

[ref_A9C3BRCS] IFA (Institut für Arbeitsschutz der Deutschen Gesetzlichen Unfallversicherung) (2023) TEPP. GESTIS-Stoffdatenbank. https://gestis.dguv.de/data?name=032770&lang=en, accessed 31 Aug 2023

[ref_XLYI8UP7] Lewalter J (1995) Acetylcholinesterase inhibitors. BAT Value Documentation, 1986. In: Greim H. Lehnert G, editors Biological Exposure Values for Occupational Toxicants and Carcinogens. Weinheim: VCH. Also available from https://doi.org/10.1002/3527600418.bb0astrinhe0002

[ref_E3YI9YZI] Weistenhöfer W, Drexler H, Hartwig A, MAK Commission (2024) Acetylcholinesterase inhibitors – Addendum: withdrawal of BAT value and continuation as BLW. Assessment Values in Biological Material – Translation of the German version from 2024. MAK Collect Occup Health Saf 9(3): Doc069. https://doi.org/10.34865/bb0astrinhe9_3ad

